# Examining factors associated with treatment completion in a community-based program for individuals with criminal justice involvement

**DOI:** 10.1186/1940-0640-10-S1-A60

**Published:** 2015-02-20

**Authors:** Lisa M Shannon, Afton Jackson, Jennifer Newell, Elizabeth Perkins, Connie Neal

**Affiliations:** 1 Sociology, Social Work and Criminology, Morehead State University, Morehead, KY, 40351, USA; 2 Department of Statewide Services, Kentucky Drug Courts, Administrative Office of the Courts, Frankfort, KY, 40601, USA

## Background

Drug court provides community-based treatment to individuals with substance abuse problems involved in the criminal justice system. The program offers comprehensive treatment as an alternative to incarceration. Past research has suggested there are sociodemographic and other personal characteristics that may impact an individual’s success in this type of program. The purpose of the current study is to examine factors associated with treatment completion among a random sample of program participants.

## Methods

A stratified random sample (n = 534) was selected from the total participants in Kentucky Drug Courts (KDC; N = 4881) from July 1, 2006, to January 1, 2011. The stratification was utilized to ensure participants were selected from all KDC sites, proportionate to the percentage the site represented of the total KDC population. Data utilized for these analyses were gathered from: the baseline assessment at program entry (a modified version of the Addiction Severity Index (ASI); [1]) and the KDC Management Information System, which contains comprehensive information on services and activities during program participation. The majority of the sample was male (60.3%) and, on average, about 30 years old (mean = 29.5; SD = 8.7).

## Results

Overall, of the 534 individuals selected as part of the stratified random sample, 36.3 percent were program graduates (n = 194). Enter binary logistic regression was utilized to examine factors associated with program completion. There were several variables positively associated with program completion including: increased age (p < .001), reporting race as Caucasian (p = .015), being single/never married (p = .004), no past history of alcohol/drug treatment (p = .019), not reporting a pension for psychiatric disability (p = .007), no referrals to short-term residential treatment (p = .033), and not having any programmatic sanctions (p = .002). There were several variables negatively associated with program completion including: having less than a high school diploma (p = .038), being unemployed (p = .011), no referrals to outpatient substance abuse treatment (p < .001), and number of positive drug tests (p = .019).

## Conclusions

Understanding factors associated with program completion is important for multiple levels of programming. First, identifying characteristics of individuals most associated with program graduation can help with targeting screening and program assessment. Further, identifying characteristics of individuals most associated with program noncompliance can help with identifying individuals most at risk within the program to develop appropriate treatment and service planning.

## References

[B1] McLellan A Luborsky L Woody G O'Brien C An improved diagnostic evaluation instrument for substance abuse patients J Nervous Mental Disorders 1980 168 26 33 10.1097/00005053-198001000-00006 7351540

